# Whole-exome sequencing identifies two novel *ALMS1* mutations in Indian patients with Leber congenital amaurosis

**DOI:** 10.1038/s41439-021-00143-z

**Published:** 2021-03-29

**Authors:** Natarajan N. Srikrupa, Sarangapani Sripriya, Suriyanarayanan Pavithra, Parveen Sen, Ravi Gupta, Sinnakaruppan Mathavan

**Affiliations:** 1 SNONGC Department of Genetics and Molecular Biology, Vision Research Foundation, Sankara Nethralaya, Chennai, India; 2 School of Chemical and Biotechnology, SASTRA deemed to-be University, Thanjavur, India; 3 Shri Bhagwan Mahavir Vitreoretinal Services, Medical Research Foundation, Sankara Nethralaya, Chennai, India; 4 MedGenome Labs Pvt. Ltd., Bangalore, India

**Keywords:** Genetics research, Genetic testing

## Abstract

Leber congenital amaurosis (LCA) is a severe autosomal recessive retinal degenerative disease. The current study describes exome sequencing results for two unrelated Indian LCA patients carrying novel nonsense p.(Glu636*) and frameshift p.(Pro2281Leufs*63) mutations in the *ALMS1* gene. Although *ALMS1* gene mutations are associated with Alstrom syndrome (AS), the current patients did not exhibit typical syndromic features of AS. These data suggest that *ALMS1* should be included in the candidate gene panel for LCA to improve diagnostic efficiency.

Leber congenital amaurosis (LCA) is one of the most severe forms of hereditary retinal dystrophy causing early infantile blindness. Affected infants show signs of night blindness, roving/pendular nystagmus, photophobia, and digito-ocular signs that are characterized by a nonrecordable electroretinogram (ERG)^1^. Accounting for 10–20% of childhood blindness, LCA is clinically heterogeneous; indeed, it is reported as an isolated clinical entity as well as in certain syndromes, such as Joubert syndrome^2^, thiamine-responsive megaloblastic anemia^3^, Senior-Loken syndrome^4^, and Batten’s disease^5^. Genetically, LCA is predominantly inherited as an autosomal recessive disease and rarely as an autosomal dominant disease. To date, mutations in at least 25 genes have been identified as causing LCA^6^. More recently, biallelic *ALMS1* gene mutations have been shown to be associated with nonsyndromic LCA^7^.

The *ALMS1* gene (2p13)^8^ encodes a basal body and centrosome-associated protein found in ciliated cells. The ALMS1 protein is involved in processes such as microtubule organization, cilium formation and maintenance, extracellular matrix production, cell migration, intraciliary transport, and cell cycle regulation^9^. This ciliary protein is reportedly expressed in multiple tissues, and as such, improper translation of *ALMS1* may result in Alstrom syndrome characterized by cone-rod dystrophy (CRD), obesity, type 2 diabetes mellitus, cardiomyopathy, hearing loss, and multiple organ failure^10^. Thus far, *ALMS1* (NM_015120) is the only gene associated with Alstrom syndrome (AS).

A proper clinical evaluation along with relevant genetic molecular testing for pathogenic disease-causing variants are necessary for a conclusive and definitive diagnosis of retinal dystrophies such as LCA, retinitis pigmentosa (RP), and CRD. We report herein two novel homozygous pathogenic genetic variants in the *ALMS1* gene in two unrelated Indian families presenting with clinical features similar to LCA, without extraocular phenotypes.

Consultation with the Vitreo Retinal Services of a tertiary eye care center was sought for the patients, who underwent complete ophthalmic and clinical evaluation, including documentation of family history, birth history, refraction, visual acuity testing, retinal examination, nystagmus, photophobia, oculo-digital signs, full-field electroretinogram (ffERG), fundus photography, and optical coherence tomography (OCT). This study was approved by the institutional Ethics Committee, and informed consent was obtained from the patients’ families. Peripheral blood samples were collected from the proband and family members, and genomic DNA was extracted using a NucleoSpin Blood XL kit (Macherey-Nagel, GmbH, Germany). In our previous study, we performed targeted resequencing of 20 candidate genes in 92 Indian LCA patients (*AIPL1, CABP4, CEP290, CRB1, CRX, GUCY2D, IQCB1, IMPDH1, KCNJ13, LCA5, LRAT, MERTK, NMNAT1, OTX2, RD3, RDH12, RPE65, RPGRIP1, SPATA7*, and *TULP1)* using an Agilent HaloPlex target enrichment assay^11^. The probands (case 1 and case 2) were negative for disease-causing pathogenic variants when screened by targeted resequencing. Hence, samples from the proband along with one parent were subjected to whole-exome sequencing (WES) using the Agilent SureSelectXT Human All Exon V5 + UTRs enrichment kit and sequenced using an Illumina HiSeq 2500 at 80–100x depth.

We obtained 8 Gb data per sample. The pipeline for processing the data involved a Burrows-Wheeler Aligner (BWA) for mapping to the GRCh37/hg19 genome build, and GATK-lite was used for realignment and recalibration of the obtained reads after duplicate removal. Variant calling was performed using the GATK-lite Unified Genotype caller and annotated using VariMAT (internal data analysis pipeline curated by MedGenome Labs, Bangalore).

Only Q30 data were considered for further annotation. The annotated variants were cross-checked with open databases such as dbSNP (v.2.0, Build 153), GenomAD, LOVD, 1000 Genomes Project, and Ensemble Variation Table to filter variants with MAF ≤ 0.01. As LCA is predominantly an autosomal recessive disease, data for the proband and the respective parent were compared to analyze variants that were found to be homozygous in the proband and heterozygous in the parent and possible compound heterozygous in the proband(s). Heterozygous variants of known dominant and recessive gene(s) [Supplementary Table 1] were also considered and ruled out after segregation and phenotypic correlation.

For Sanger sequencing of the identified variants in exon 8, primers were designed using the *ALMS1* transcript ENST00000264448.6 using Primer 3 (v.0.4.0), as follows: for g.73675557 G>T, forward 1-5′TGACCAGACAACTGGCATGTC3′ and reverse 1-3′GACTGTCTGCTAAGTCCTGTG5′; for g.73680491delT, forward 2-5′CTCAGGCTGATGACAGAGTTG3′ and reverse 2-3′GGTGTAGTGGAACCATTGGG5′. PCR was standardized using a touchdown protocol of 63.5–56.5 (−0.5 °C) for g.73675557 G>T and 59.5 °C annealing temperature for g.73680491delT with a reaction mixture comprising 10 pmol/µl primers (forward and reverse), 1X Taq buffer, 50 ng DNA template, 0.5 mM dNTPs, and 0.5 U Taq polymerase. The PCR products were purified by Exo-SAP and cycle sequenced using a Big dye Terminator v3.1 kit (Applied Biosystems, USA). The purified products were sequenced using an ABI 3500 *Avant* genetic analyzer.

Regarding case 1, the child was first seen at 18 months of age with a history of profound visual loss (counting fingers [CF] at one meter in both eyes) and nystagmus since 6 months of age. The pedigree, as indicated in Fig. 1A shows an isolated family history. The refractive error was +3 D sphere in both eyes. Full-field ERG was performed under general anesthesia and inferred to be unrecordable under both scotopic and photopic conditions (Fig. 1B). Fundus examination was performed under anesthesia and revealed disc pallor with marked arteriolar attenuation, a salt and pepper appearance of the retina, and sparse pigmentation (Fig. 1C). The macula showed a dull reflex, but no macular scarring, pigmentation, or bull’s eye lesion was seen. At a follow-up visit at 12 years of age, we observed that the vision of the child was the same as before, and ERG continued to be unrecordable. The child was cooperative for OCT at this visit, which revealed the presence of a shallow foveal dip with thinning of the outer nuclear and ellipsoid layers (Fig. 1D). This presentation is similar to LCA with extinguished responses on ffERG in the first year of life. Whole-exome sequencing for the proband and unaffected father identified homozygous novel nonsense mutation (g.73675557 G>T; c.1906G>T (NM_015120); p.(Glu636*)), leading to premature truncation of the ALMS1 protein (Fig. 1E). Sanger sequencing identified the parents and unaffected siblings to be heterozygous (Fig. 1F) for the variant.

**Fig. 1:**
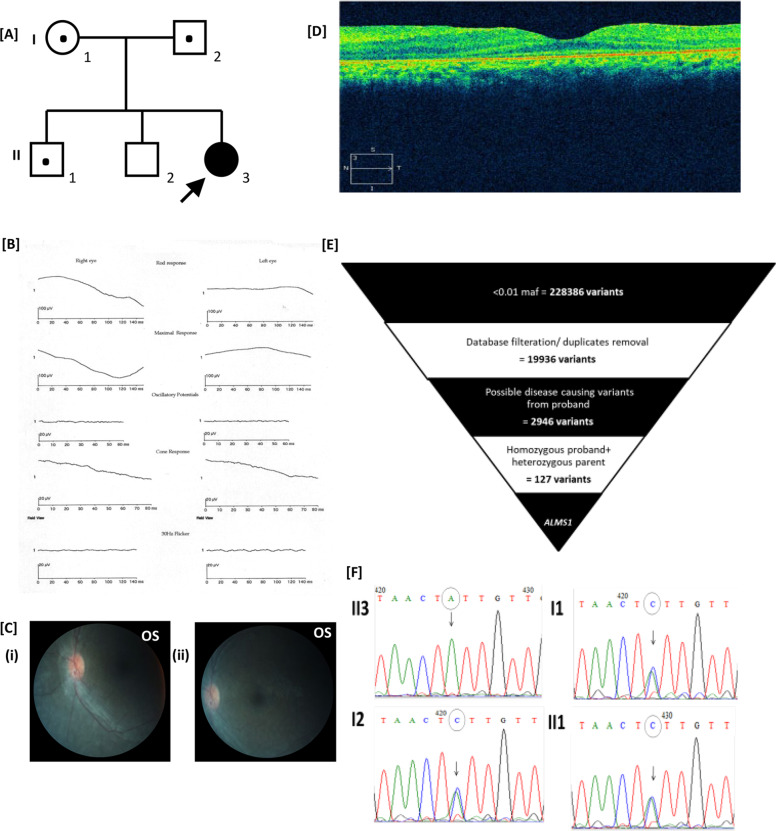
Clinical and phenotypic documentation in case 1. [**A**] Family pedigree: affected status is indicated by a shaded circle, and carrier status is indicated by a dot (●). [**B**] ERG is nonrecordable. [**C**] Fundus photograph of case 1 (i) showing a near normal looking macula (ii) with RPE granularity in the mid periphery. [**D**] OCT shows a preserved foveal dip with thinning of the outer nuclear and ellipsoid layer. [**E**] The analysis, validation, and identification of WES data. [**F**] Electrophoretogram showing the reverse-strand sequence of the g.73675557â€‰G>T; c.1906G>T; p.(Glu636*) variant, homozygous (**II3**) in the proband and heterozygous in the (**I1**) mother, (**I2**) father, and (**II1**) unaffected sibling.

Case 2 was brought to the clinic at 2 and half years of age, and the parents described observing poor vision in the child since the first year of life. The pedigree indicated no family history (Fig. 2A). BCVA was very poor in the proband, with both eyes exhibiting only fixation and light following. The child had nystagmus and photophobia at presentation, with bilateral hyperopia of +8.00 D. Nonrecordable photopic responses for both 3.0 and 30 Hz flickers were obtained by ffERG. Dark-adapted (DA) responses were extinguished for the DA 0.01 flash but were recordable, though with markedly reduced amplitudes for the DA 3.0 flash. These findings are suggestive of severe and widespread effects of the rod and cone system, with the cone system being more severely affected (Fig. 2B). Fundus examination revealed the presence of disc pallor with arteriolar attenuation, widespread peripheral granularity, and early macular involvement in the form of macular pigment mottling with bony spicule pigmentation (Fig. 2C). The child at this age was not cooperative for OCT. Molecular testing by WES identified a novel homozygous deletion variant (Fig. 2D), g.73680491delT; c.6840delT (NM_015120), leading to a frameshift and thereby truncated protein (p.Pro2281Leufs*63). The deletion variant segregated in the parents, with a heterozygous genotype (Fig. 2E).

**Fig. 2:**
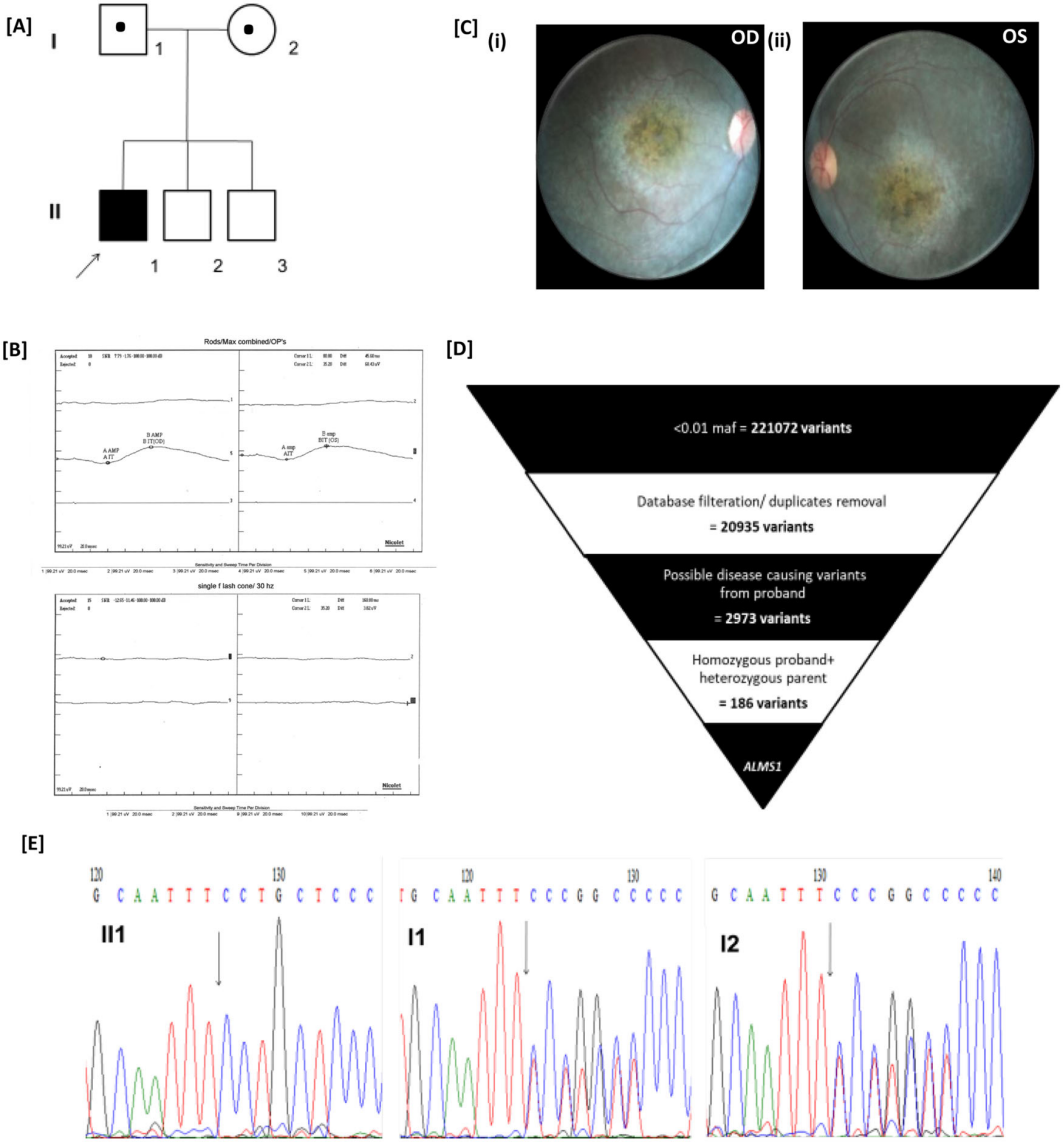
Clinical and phenotypic documentation in case 2. [**A**] Family pedigree: affected status is indicated by a shaded square and carrier status by a dot (●). [**B**] ERG shows severely reduced rod and cone responses, with cone responses almost extinguished. [**C**] Fundus photograph of case 2 (i & ii): right and left eye of the child showing a large macular scar, arteriolar attenuation, and RPE granularity. [**D**] The analysis, validation, and identification of WES data. [**E**] Electrophoretogram showing the g.73680491delT; c.6840delT; (p. Pro2281Leufs*63) variant, homozygous (**II1**) in the proband and heterozygous in the (**I1**) father and (**I2**) mother.

Control samples (*N* = 100) exhibited the wild-type genotype for both variants by Sanger sequencing. The variants were not found in open databases, and in silico tools such as Mutation Taster^12^ and CADD^13^ predicted them to be disease-causing. Both variants might lead to nonsense-mediated decay (NMD) because they are located ~50–55 nucleotides upstream of the last exon–exon junction^14^; nevertheless, characterization studies are essential to prove this functionality. The variants are classified as strongly pathogenic according to ACMG guidelines (PVS1, PM-1, 2, 4, PP1, 3)^15^.

In conclusion, we identified two novel pathogenic variants in exon 8 of the *ALMS1* gene in patients with LCA but without any symptoms of AS. Similar to the current study, WES has revealed compound heterozygous null mutations or homozygous mutations in *ALMS1* in LCA or early-onset severe cone-rod dystrophy cases without extraocular abnormalities^16^, thus indicating *ALMS1* as a candidate gene for isolated LCA/early-onset retinal diseases other than AS. As LCA is congenital, it could be the primary manifestation of AS, and careful clinical examination along with confirmatory genetic testing is necessary to elucidate the underlying gene, especially in the absence or late onset of other syndromic features. The manifestations of AS phenotypes begin as early as a few days after birth in retinal degeneration or cardiomyopathy and later in adulthood/the second decade, such as the indication of type 2 diabetes and renal or hepatic abnormality^17^.

Analysis of genotype and phenotype correlation in AS cases harboring a mutation in exon 8 of the *ALMS1* gene has suggested an association between exon 8 mutations and a normal^18^ or delayed renal disease phenotype^19^. This emphasizes the phenotypic heterogeneity for this protein depending on the mutation. In the current study, except for delayed milestone presentation in case 2, the patients did not show any systemic features specific for AS (current ages are 19 yrs for case 1 and 18 yrs for case 2). Nevertheless, biochemical tests for hepatic and renal problems or to diagnose early diabetes were not performed. Annual monitoring of the cardiac, hepatic, and renal phenotypes of such patients will be helpful for differential diagnosis, early treatment, better disease management, and rehabilitation of future cases.

## Supplementary information

Supplementary Table 1
