# Supplementary material for: Whole-exome sequencing identifies two novel ALMS1 mutations in Indian patients with Leber congenital amaurosis
Source: Hum Genome Var. 2021 Mar 29;8:12. doi: 10.1038/s41439-021-00143-z (PMC8007799; doi:10.1038/s41439-021-00143-z)
Supplement: Supplementary file 1 — Supplementary Table 1 [file 41439_2021_143_MOESM1_ESM.docx]

Supplementary Table 1:

Other potential causal variants identified in both the cases by WES.

| **CASE 1** | | | | | | | | |
| --- | --- | --- | --- | --- | --- | --- | --- | --- |
| **CHROMOSOME**  **NUMBER** | | **GENE_ NAME** | **VARIANT CLASS** | **ZYGOSITY** | | **GENOME_ CHANGE** | **CDNA_ CHANGE** | **PROTEIN_ CHANGE** |
| chr16 | | *ABCC6* | Missense | Heterozygous | | g.16255404A>G | c.3524T>C | p.Val1175Ala |
| chr14 | | *RPGRIP1* | Missense | Heterozygous | | g.21756185T>C | c.50T>C | p.Ile17Thr |
| chr12 | | *CEP290* | Frameshift-  Ins | Heterozygous | | g.88512304A>AT | c.832dup | p.Ile278Asnfs*20 |
| chr4 | | *LRIT3* | Missense | Heterozygous | | g.110791030A>T | c.1125A>T | p.Arg375Ser |
| chr1 | | *USH2A* | Missense | Heterozygous | | g.216371836C>A | c.3902G>T | p.Gly1301Val |
| **CASE 2** | | | | | | | | |
| chr18 | *LAMA1* | | Missense | Heterozygous | g.7044753G>A | | c.944C>T | p.Pro315Leu |
| chr10 | *CDH23* | | Missense | Heterozygous | g.73326643G>C | | c.709G>C | p.Glu237Gln |
| chr11 | *MYO7A* | | Missense | Heterozygous | g.76919766A>G | | c.5852A>G | p.Gln1951Arg |
| chr1 | *USH2A* | | Missense | Heterozygous | g.216172376A>C | | c.6510T>G | p.Ser2170Arg |
